# Differential effects of *Mycobacterium bovis* - derived polar and apolar lipid fractions on bovine innate immune cells

**DOI:** 10.1186/1297-9716-43-54

**Published:** 2012-06-27

**Authors:** Chris Pirson, Gareth J Jones, Sabine Steinbach, Gurdyal S Besra, H Martin Vordermeier

**Affiliations:** 1 TB Research Group, Animal Health and Veterinary Laboratories Agency – Weybridge, New Haw, Surrey, Addlestone, KT15 3NB, United Kingdom; 2 School of Biosciences, University of Birmingham, Edgbaston, Birmingham, B15 2TT, United Kingdom

## Abstract

Mycobacterial lipids have long been known to modulate the function of a variety of cells of the innate immune system. Here, we report the extraction and characterisation of polar and apolar free lipids from *Mycobacterium bovis* AF 2122/97 and identify the major lipids present in these fractions. Lipids found included trehalose dimycolate (TDM) and trehalose monomycolate (TMM), the apolar phthiocerol dimycocersates (PDIMs), triacyl glycerol (TAG), pentacyl trehalose (PAT), phenolic glycolipid (PGL), and mono-mycolyl glycerol (MMG). Polar lipids identified included glucose monomycolate (GMM), diphosphatidyl glycerol (DPG), phenylethanolamine (PE) and a range of mono- and di-acylated phosphatidyl inositol mannosides (PIMs). These lipid fractions are capable of altering the cytokine profile produced by fresh and cultured bovine monocytes as well as monocyte derived dendritic cells. Significant increases in the production of IL-10, IL-12, MIP-1β, TNFα and IL-6 were seen after exposure of antigen presenting cells to the polar lipid fraction. Phenotypic characterisation of the cells was performed by flow cytometry and significant decreases in the expression of MHCII, CD86 and CD1b were found after exposure to the polar lipid fraction. Polar lipids also significantly increased the levels of CD40 expressed by monocytes and cultured monocytes but no effect was seen on the constitutively high expression of CD40 on MDDC or on the levels of CD80 expressed by any of the cells. Finally, the capacity of polar fraction treated cells to stimulate alloreactive lymphocytes was assessed. Significant reduction in proliferative activity was seen after stimulation of PBMC by polar fraction treated cultured monocytes whilst no effect was seen after lipid treatment of MDDC. These data demonstrate that pathogenic mycobacterial polar lipids may significantly hamper the ability of the host APCs to induce an appropriate immune response to an invading pathogen.

## Introduction

Bovine tuberculosis (BTB), caused by *Mycobacterium bovis* (*M. bovis*), is a zoonotic disease of significant economic, animal and public health burden. Consumption of raw or unpasteurised animal products and contact with infected carcasses plays a large role in zoonotic *M. bovis* infection of humans in Africa and South America [1,2]. Yet this is not a problem solely associated with poverty and less developed countries as evidenced by a recent report that up to 45% of all TB infected children in San Diego were caused by *M. bovis*[3]. The incidence of BTB in cattle in Great Britain (GB) has undergone steady and continual increase since 1998 despite the implementation of control measures, possibly due to the presence of a wildlife reservoir [4]. Within GB, BTB has spread drastically since the Foot & Mouth disease outbreak in 2001 with the annual number of animals slaughtered rising from a mean of 7116 animals between 1998 and 2001 to an annual mean of 26 277 cases between 2002 and 2010 inclusive [5].

The first point of contact between *M. bovis* and its host is likely to be interaction between receptors of its sentinel cells of the innate immune system such as macrophages or dendritic cells (DC) and the surface expressed molecules of the bacilli, the majority of which are lipid in nature [6,7]. Recognition of mycobacterial lipids by macrophages has been demonstrated via the mannose receptor, complement receptors, scavenger receptors and CD14 [8-10]. Furthermore, various lipids from *M. tuberculosis* have been shown to ligate TLR2 on human macrophages [11]. DC are known to possess and utilise these same receptors as well as other, structurally related molecules such as the DC specific C - type lectin (DC - SIGN) which have also been heavily implicated in immune recognition of the bacilli [12-14].

Many lipids have been implicated in mycobacterial virulence which are not found in other bacterial genera. Lipomannan (LM), lipoarabinomannan (LAM), the phosphatidylinositol mannosides (PIMs), the cord factors trehalose mono- and dimycolate (TMM and TDM) and the phthiocerol dimycocerosates (PDIMs) are all surface bound mycobacterial lipids capable of modulating innate immunity [15-17]. Recently, newly identified lipids, such as monomycolyl glycerol (MMG), have been shown to modulate host immunity [18] and hypervirulence [19]. However, little data exist which describe the effect of *M. bovis* derived lipid on bovine innate cells. Previously published work usually relies on non - pathogenic mycobacterial species or makes use of an animal model rather than the pathogen’s natural host. For example, work performed by Hope et al. uses bovine monocyte-derived DC (MDDC) but these cells are stimulated with a synthetic lipopeptide [20] rather than a *M. bovis*-specific lipid antigen. Additionally, although Reed et al. demonstrated that blockage of synthesis of the phenolic glycolipid (PGL) correlated with increased secretion of TNF-α, IL - 6 and IL - 12 by the host, and removed the “hyperlethal” phenotype displayed by the bacilli, this was only shown in the murine model [19].

Thus, to assess host immune responses to its natural pathogen’s lipid constituents, lipids were extracted from virulent *M. bovis* AF 2122/97 and used to stimulate various bovine innate immune cells isolated from live, TB free, cattle. Cellular responses were evaluated by measuring cytokine production, alterations in cell surface molecules and induction of T-cell proliferation.

## Materials and methods

### Preparation of bacterial isolates for lipid extraction

Bacterial isolates were grown in Middlebrooks 7H9 medium as previously described [21].

Briefly, bacterial cells were grown in 100 mL volumes in rolling culture flasks inoculated with 1 mL of a starting culture. At mid ‒ log phase, cultures were decanted into sterile tubes and pelleted before being washed twice in sterile water. Finally, pellets were resuspended in 5 mL sterile water and heat killed in a water bath at 80 °C to 90 °C for between 1 and 2 h and finally freeze dried.

### Extraction of crude free mycobacterial lipid

The extraction of mycobacterial lipid has been previously described [22]. Briefly, freeze dried bacterial cells were suspended in methanolic saline before an equal volume of petroleum ether was added and the mixture stirred for 12 to 16 h. Cells were pelleted by centrifugation (7000 *g* for 10 min) and the non‒aqueous phase containing the apolar lipids was removed and stored. An equal amount of petroleum ether was added to the aqueous lower phase and the mixture stirred for 2 h before being centrifuged (7000 *g* for 10 min) and the non‒aqueous layer removed and pooled with the first. These non-aqueous petroleum ether extracts were dried using a rotary evaporator with cold finger condenser and the lipid transferred to a pre‒weighed glass tube in 4 : 1 CHCl_3_ : CH_3_OH. Evaporation of the CHCl_3_ : CH_3_OH was achieved using a heating block and N_2_ gas stream and the tube weighed to determine the mass of apolar lipids extracted.

Extraction of polar lipids was performed by adding CHCl_3_, CH_3_OH and 0.3% aqueous NaCl in a 9 : 10 : 3 ratio to the cell pellet. The mixture was stirred for 12 to 16 h before being passed through 2 Whatman # 91 filters. Once dried, the cells were recovered from the filter papers and re‒extracted twice using a 5 : 10 : 4 mixture of CHCl_3_, CH_3_OH and 0.3% NaCl. After a final filtration to remove the cells, equal volumes of CHCl_3_ and 0.3% NaCl were added and the mixture stirred for one hour, after which the aqueous phase containing the polar lipids was removed and dried in a rotary evaporator. Final polar lipid mass was ascertained as described for the apolar petroleum ether extracted lipid fraction.

### Analysis of lipid fractions by 2D Thin Layer Chromatography (TLC)

**Figure 1:**
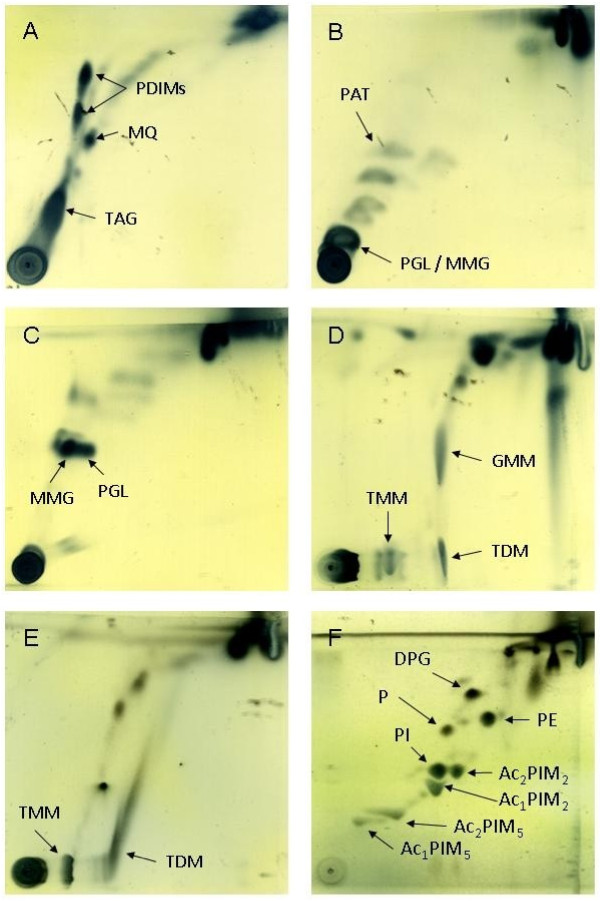
**2D TLC analysis of crude, free lipids extracted from M. bovis AF 2122/97 and stained with MPA.A - D**: Apolar fraction analysed with TLC systems A, B, C and D. **E** and **F**: Polar fraction analysed with systems D and E. PDIM - phthiocerol dimycocerosate; MQ - menaquinone; TAG - triacyl glycerol; PAT - pentacyl trehalose; PGL - phenolic glycolipid; MMG - monomycolyl glycerol; TMM - trehalose monomycolate; TDM - trehalose dimycolate; GMM - glucose monomycolate; DPG - diphosphatidyl glycerol; PE - phosphatidyl ethanolamine; PIM - phosphatidylinositol mannosides (integers denote number of mannoside or acyl groups); PI - phosphatidyl inositol; P - phospholipid.

Aluminium backed silica gel 60 F_254_ TLC plates (Fisher Scientific, Loughborough, Leics, UK) were cut into approximately 6 cm squares and 100 μg of lipid extract was spotted onto the plates using glass micro - capillary pipettes. Plates were dried thoroughly before being placed in TLC tanks containing appropriate solvent mixtures (systems A - E, Table 1). TLC plates were dried between each run and before staining to ensure that residual solvent was removed. Staining was performed using a 5% solution of molybdophosphoric acid (MPA) (Sigma Aldrich, Poole, Dorset, UK) in 95% ethanol (Figure 1). Stains were sprayed onto TLC plates which were subsequently charred using a hot air gun before being photographed or scanned. Identification of individual lipids was performed by comparison with previously published TLC analysis [23].

**Table 1:** **Solvent systems for TLC analysis of mycobacterial lipids (adapted from**[22]**).**

**Solvent System**	**Run Direction**	**Components**	**Runs**	**Lipids Resolved**
A	1	petroleum ether : ethyl acetate (98 : 2)	3	PDIM, TAG, MQ
	2	petroleum ether : acetone (98 : 2)	1	
B	1	petroleum ether : acetone (92 : 8)	3	AT, FA
	2	toluene : acetone (95 : 5)	1	
C	1	chloroform : methanol (96 : 4)	1	FA, GLY
	2	toluene : acetone (80 : 20)	1	
D	1	chloroform : methanol : water (100 : 14 : 0.8)	1	CF, SL, DAT
	2	chloroform : acetone : methanol : water (50 : 60: 2.5 : 3)	1	
E	1	chloroform : methanol : water (60 : 30 : 6)	1	DPG, PE PI, PIM
	2	chloroform : acetic acid : methanol : water (40 : 25 : 3 : 6)	1	

### Cattle

BTB free cattle between the ages of 6 and 36 months were obtained from herds within 4 - yearly testing parishes with no history of a BTB breakdown in the past 4 years. These animals were purchased when around 6 months old and transported to AHVLA. Whilst at AHVLA they tested negative for BTB using both the Bovigam IFNγ assay (Prionics AG, Schlieren-Zurich, Switzerland) and the single intradermal comparative cervical tuberculin test (SICCT).

### Isolation of bovine PBMC from whole blood

Whole blood was mixed in equal amounts with sterile HBSS containing 10 U mL^-1^ heparin. This mixture was overlaid onto Histopaque 1077 (Sigma Aldrich) and centrifuged at 800 *g* for 40 min. The PBMC interface was removed using a pastette and washed twice in HBSS containing heparin. Live cells were identified via trypan blue exclusion and enumerated using a haemocytometer.

### Isolation of CD14^+^ monocytes from bovine PBMC

PBMC were suspended in 80 μL of MACS rinsing buffer per 10^7^ cells before the addition of 10 μL of MACS anti - CD14 MicroBeads (Miltenyi Biotec, Bisley, Surrey, UK) per 10^7^ cells. After a 15 min incubation at +4 °C on a rotator, cells were pelleted and resuspended in 500 μL per 10^8^ cells and passed through MACS LS columns as per the manufacturer’s instructions.

The CD14^+^ fraction was counted and cells diluted to 1.5 × 10^6^ mL^-1^ in cell culture medium (RPMI 1640 containing 25 mM HEPES, 10% FCS, 1% NEAA, 5 × 10^-5^ mM β2 - mercapto-ethanol, 100 U mL^-1^ penicillin and 100 μg mL^-1^ streptomycin [Gibco Life Technologies, Paisley, UK]).

### Generation of bovine cultured monocytes and MDDC

CD14^+^ monocytes were plated in 1 mL volumes at 1.5 × 10^6^ mL^-1^ in 24 well plates (Nunc Nunclon, Roskilde, Denmark) before adding either 1000 U mL^-1^ equine GM-CSF (Kingfisher Biotech, St Paul, MN, USA) (cultured monocytes) or 1000 U mL^-1^ equine GM-CSF and 4 ng mL^-1^ bovine IL-4 (AbD-Serotec, Kidlington, Oxon, UK ) (MDDC). Cells were cultured at 37 °C + 5% CO_2_ for 3 days [20], following which they were harvested, re-plated at 1.5 × 10^6^ mL^-1^ in fresh cell culture medium and the appropriate volume of lipid solution was added. Cells were cultured for a further 12 to 16 h before supernatants were collected and cells harvested for subsequent flow cytometric analysis.

### Preparation of lipid antigen suspensions

Suspensions of all lipid antigens were prepared in an aqueous phase for use in cell culture experiments after first removing any CHCl_3_ : CH_3_OH by evaporation using an N_2_ gas stream. Cell culture medium was added to the dried lipid and the mixture subjected to 2 cycles of heating at 80 °C and then sonication for 5 min. Apolar and polar lipids were used to stimulate cells in vitro at 20 μg mL^-1^ for 12 to 16 h. These conditions were shown to be optimal in previous experiments (data not shown).

### Measurement of cytokine production

Culture supernatants were assayed for cytokine levels using the MSD multiplex platform (Meso Scale Discovery, Gaithersburg, MD, USA) as previously described [24,25]. Briefly, supernatants were analysed using a custom multiplex electrochemiluminescent system which allows simultaneous detection of IL-1β, IL-6, IL-10, IL-12, MIP-1β and TNF-α (Meso Scale Discovery). Multiplex 96 well plates were supplied with target capture antibodies spotted onto 6 separate carbon electrodes in each well (anti-bovine TNF-α [Endogen, Rockford, IL, USA]; anti-bovine IL-10 and anti-bovine IL-12 [AbD-Serotec]; anti-bovine IL-1β, anti-bovine IL-6 and cross-reactive anti-human MIP-1β [Meso Scale Discovery]). Plates were blocked with MSD assay buffer for 30 min at room temperature before the addition of samples or standards for 1 h at room temperature. Recombinant standard controls (Meso Scale Discovery) were prepared by serial dilution. After incubation, plates were washed and combined biotinylated secondary detector antibodies were added for a further hour. Finally, plates were washed, loaded with MSD read buffer and analysed using an MSD Sector Imager 6000.

### Cell labelling and analysis by flow cytometry

Cultured cells were suspended in PBS and labelled with the live / dead indicator ViViD (Invitrogen Life Technologies, Paisley, UK) before being transferred to a 96 well plate and washed using 150 μL MACS rinse buffer. Cells were stained for 15 min using either anti-bovine CD14 (ccG33; Institute for Animal Health; 1:50 dilution), anti-equine MHCII (MCA1085; AbD Serotec; 1:50 dilution), anti-bovine CD40 (IL-A156; cell supernatant; AHVLA; 1:10 dilution), anti-bovine CD80 (IL - A159; cell supernatant; AHVLA; 1:10 dilution), anti-bovine CD86 (IL-A190; cell supernatant; AHVLA; 1:10 dilution), anti-bovine CD1b (CC14; AbD Serotec MCA831G; 1:10 dilution) or an IgG1 isotype control (Av20; Institute for Animal Health; 1:50 dilution). Labelled cells were washed using 150 μL MACS rinse buffer and secondary labelling was performed using a 1:400 dilution of anti-IgG1 conjugated to R-Phycoerythrin (R-PE) (Invitrogen; P21129) in 50 μL volumes for 10 min. After incubation, cells were washed by the addition of 150 μL PBS, pelleted and resuspended in 100 μL of 2% paraformaldehyde (Cytofix; BD Biosciences, Oxford, Oxon, UK) for at least 30 min at 4 °C before analysis on a CyAn ADP analyser. For capture and analysis, initial gating was on single, ViViD^lo^ (live) cells into a subsequent small cell/lymphocyte exclusion gate.

### One way mixed lymphocyte reaction

Bovine MDDC and cultured monocytes were prepared from 1 animal as described above. Following 3 days in culture, cells were pulsed with lipid antigen overnight before being enumerated, washed to remove any cytokines and lipid from the media and incubated at 10^7^ cells mL^-1^ in the presence of Mitomycin C at 100 μg mL^-1^ for 30 min at 37 °C + 5% CO_2_. Lipid pulsed, Mitomycin C treated MDDC or cultured monocytes were cultured in 1 mL at 37 °C + 5% CO_2_ at 2 × 10^5^ with 1 × 10^5^ PBMC isolated from a second, allogeneic animal. After 5 days, cells were pulsed overnight with 1 μCi well^-1^ of ^3^ H-thymidine before being harvested using a Harvester 96 Mach III (TomTec Inc, Hamden, CT, USA). Lymphocyte proliferation was assessed by the increased cellular incorporation of ^3^ H-thymidine which was measured using a MicroBeta^2^ 2450 (Perkin Elmer, Waltham, MA, USA).

### Data and statistical analysis

All data representation and statistical analysis was performed using GraphPad Prism version 5.04 and GraphPad InStat version 3.06 (GraphPad Software, La Jolla, CA, USA). Flow cytometric data was analysed using repeated measures ANOVA with a Bonferroni multiple comparisons post test. Cytokine profile analysis was performed using a Friedman non-parametric repeated measures ANOVA with Dunns multiple comparisons test.

## Results

### Extraction and analysis of lipid from AF 2122/97

In order to identify individual lipid components within the polar and apolar fractions, lipids extracted from the *M. bovis* reference strain (AF 2122/97) were subjected to 2D TLC analysis using solvent systems of increasing polarity and subsequently stained with MPA. Analysis of the apolar fraction using the least polar TLC system (Figure 1a) identified the presence of phthiocerol dimycocerosates (PDIMs), menaquinone (MQ) and triacyl glycerol (TAG). System B (Figure 1b) revealed that the apolar fraction also contained pentacyl trehalose (PAT), phenolic glycolipids (PGL) and monomycolyl glycerol (MMG). System C (Figure 1c) allowed further resolution of both MMG and PGL. The most polar system used to analyse the apolar fraction (system D, Figure 1d) identified both trehalose monomycolate (TMM) and dimycolate (TDM; cord factor) as well as glucose monomycolate (GMM).

TLC analysis of the polar lipid fraction using system D showed the presence of TMM and TDM (Figure 1e) while the most polar solvent system (E, Figure 1f) enabled identification of the most polar lipids, which included diphosphatidyl glycerol (DPG), phosphatidyl ethanolamine (PE), phosphatidylinositol mannosides (PIMs, integers denote number of mannoside or acyl groups), phosphatidyl inositol (PI) and an unknown phospholipid (P).

### Cytokine responses to *M. bovis* - derived lipids

**Figure 2:**
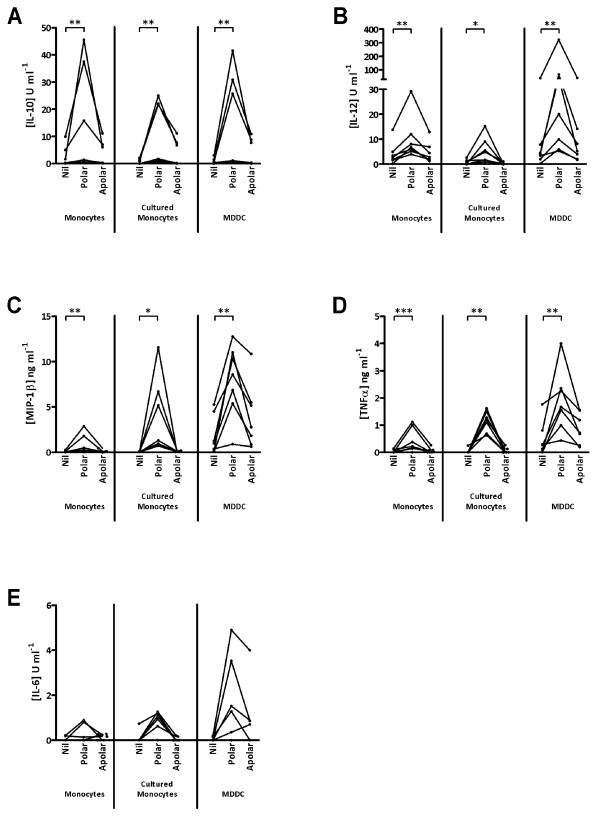
**IL - 10 (A), IL - 12 (B), MIP - 1β (C), TNFα (D) & IL - 6 (E) production by monocytes and cultured cells in response to stimulation with crude lipid fractions.** Points represent mean responses from duplicate wells for each of 7 animals tested. Lines indicate that cells were derived from the same animal; * 0 < 0.05; ** *p* < 0.01; *** *p* < 0.001.

Innate immune cells are known to produce cytokines in response to appropriate antigenic stimuli. In order to assess the effect of *M. bovis* - derived lipids on 3 types of bovine innate cells (freshly isolated monocytes, cultured monocytes and MDDC), cytokine production was measured after stimulation with the lipid fractions (Figure 2). The cytokines investigated included IL-10, IL-12, TNF-α, MIP-1β, and IL-6.

Significantly increased IL-10 secretion was seen from all 3 cell types following stimulation with the polar lipid fraction (Figure 2a). Strong IL-10 responses were seen for 3 animals, while only modest increases were noted for the remaining cattle (Figure 2a). In contrast, little or no significant increase in IL-10 production was seen following stimulation with the apolar lipid fraction, although apolar lipids induced some IL-10 production by cultured monocytes and MDDC from 3 animals. All responses to apolar lipids were by far lower than those induced by the polar lipid fraction.

**Figure 3:**
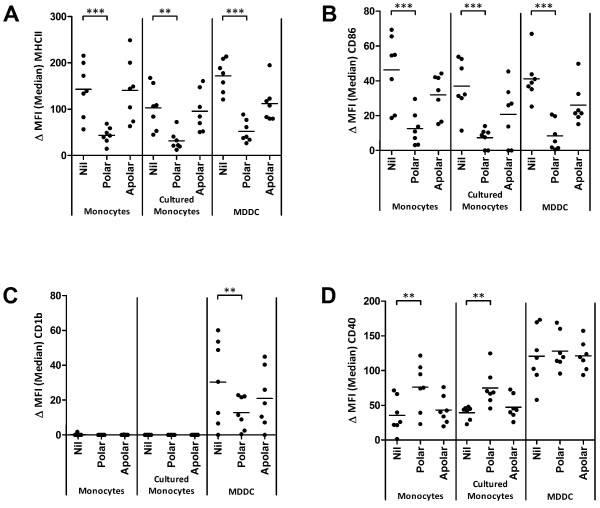
**Surface expression of MHCII (A), CD86 (B), CD1b (C) and CD40 (D) on cultured cells and fresh monocytes after exposure to crude polar and apolar lipid fractions.** A single point represents the median fluorescence intensity of the specific stain after subtraction of an isotype control (ΔMFI) for each of 7 animals tested; ** *p* < 0.01; *** *p* < 0.001.

IL-12 levels in the culture supernatants were measured simultaneously and the results are shown in Figure 2b. Stimulation with the polar lipid fraction induced a large increase in IL-12 production by MDDC, while lower responses were also observed from monocyte and cultured monocyte populations. In contrast to the polar lipids, stimulation with the apolar lipid fraction resulted in minimal increases in IL-12 production by monocytes, cultured monocytes or MDDC.

Levels of MIP-1β were also found to be significantly increased after exposure to the polar lipid fraction with no significant increase seen after apolar lipid stimulation (Figure 2c). Both cultured monocytes and MDDC produced noticeably more MIP-1β than fresh monocytes, with MDDC from 6, and cultured monocytes from 3, of the 7 animals responding strongly (Figure 2c).

Significant increases in TNFα production were also seen, again in response to the polar lipid fraction (Figure 2d). While polar lipid treated cultured monocytes from all 7 cattle produced significant levels of TNFα, considerably more TNFα was produced by MDDC (Figure 2d). Further, the level of TNFα production was similar between fresh and cultured monocytes (Figure 2d).

The production of IL-6 (Figure 2e) followed a broadly similar pattern to that of TNFα (Figure 2d) although statistical significance was not achieved. Fresh monocytes from 2 cattle produced more IL-6 after exposure to the polar lipids, which also drove increased IL-6 production in cultured monocytes from 6 cattle (Figure 2e). Polar lipid driven IL-6 production by MDDC was noted in 5 of the 7 animals screened, with one of these animals producing more IL-6 to the apolar lipid fraction than the polar. While statistical significance was not achieved, the levels of IL-6 produced by MDDC are notably higher than from fresh or cultured monocytes (Figure 2e).

These data clearly demonstrate that the polar lipid fraction drives the production of significant amounts of IL-10, IL-12, MIP-1β and TNFα from all cell types. Furthermore, it is clear that MDDC produced more IL-12, TNFα and IL-6 than fresh or cultured monocytes and MIP-1β production is greater from both cultured monocytes and MDDC than in fresh monocytes.

### Phenotypic responses to *M. bovis* - derived lipids

Exposure of bovine antigen presenting cells to the polar lipid fraction lead to significant increases in the production of a variety of cytokines (Figure 2a-e), all of which can play important roles in directing the subsequent cell mediated response. In order to further assess the effect of *M. bovis* - derived lipids and the local cytokine milieu on these cells, analysis of the expression of key antigen presentation related molecules was assessed by flow cytometry (Figure 3).

Stimulation with the polar lipid fraction resulted in a significant reduction in the cell surface expression of MHCII on all three cell types (Figure 3a). Furthermore, MHCII expression was also lower on MDDC following stimulation with apolar lipids, although this did not achieve statistical significance (Figure 3a). CD86 expression was also significantly reduced on all three cell types following stimulation with the polar lipid fraction (Figure 3b). While there was a trend for lower CD86 expression on all three cell types following stimulation with the apolar lipid fraction, this again did not achieve statistical significance (Figure 3b). Interestingly, the lipid-specific antigen presentation molecule CD1b, which was constitutively expressed on MDDC, was not present on monocytes or cultured monocytes nor could it’s expression be modulated in these cell types by mycobacterial lipids (Figure 3c). By contrast, MDDC expressed CD1b constitutively and incubation with the polar lipid fraction resulted in a significant reduction in CD1b surface expression (Figure 3c).

Not all cell surface molecules were down-regulated following treatment with lipids. CD40 expression on both monocytes and cultured monocytes increased significantly following stimulation with the polar lipid fraction (Figure 3d), although no effect was seen on MDDC. Finally, no significant difference was seen in CD80 levels following stimulation with either the polar or apolar lipid fractions (data not shown).

In summary, these data demonstrate that *M. bovis* - derived lipids, and in particular the polar fraction, downregulate the expression of several key cell surface molecules involved in antigen presentation.

### Consequence of exposure to *M. bovis* - derived lipids

**Figure 4:**
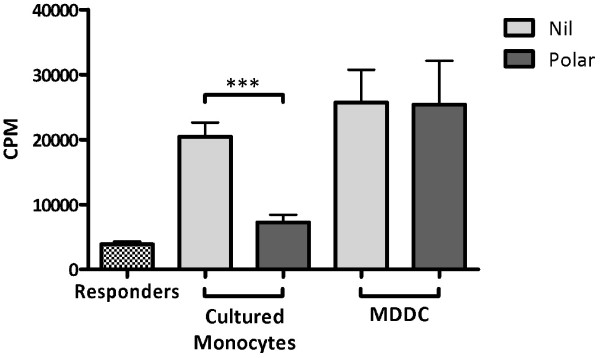
**Proliferative responses (in counts per minute [CPM]) of 1 × 10**^**5**^**PBMC after allotypic stimulation with either untreated (grey bars) or polar lipid treated (red bars) MDDC or cultured monocytes.** Bars represent the mean of triplicate wells ± standard error of the mean; *** *p* < 0.001.

To identify and assess any functional consequence of the lipid induced reduction in molecules related to antigen presentation the ability of lipid treated innate cell types to stimulate an alloreactive response was assessed. Co-culture of the responder PBMC population with either untreated MDDC or cultured monocytes resulted in a 5 - fold increase in their proliferation (Figure 4). No proliferation was noted for either MDDC or cultured monocytes in the absence of responder cells (data not shown). Polar lipid treated MDDC retained their ability to induce proliferation in the responder population despite the downregulation of important costimulatory molecules. In contrast, allo - stimulation of the responder population by polar lipid treated cultured monocytes resulted in significantly reduced proliferative responses to levels comparable with the unstimulated responder control cells (Figure 4).

## Discussion

Mycobacterial lipids have long been implicated in the interaction between the pathogen and its host. Here we describe the consequence of exposure to lipids derived from a virulent *M. bovis* on the innate immune cells of cattle.

Extraction of mycobacterial lipids and their subsequent analysis by 2D TLC has been previously described [22], but little data exists on total lipid profiling of *M. bovis.* Previous work by Dandapat et al. [26] attempted to characterise *M. bovis* based on the expression of PGL and PDIMs, but only as a tool for identification of the organism. In Figure 1, we have applied a complete range of TLC analyses to polar and apolar lipid extracts which has allowed the identification of a broad range of characteristic mycobacterial lipids including PDIMS (Figure 1a), the *M. bovis* characteristic PGL [27] (figure 1 B-C), TDM (Figure 1d-e) and PIMs (Figure 1f). As expected, no sulphoglycolipid was found (Figure 1d). Interestingly, TDM was found in both the polar and apolar extracts (Figure 1d-e). This may be related to it’s particularly amphipathic nature [28] and variable acylation states which are known to alter both immunogenicity and hydrophobicity [29,30] and may cause the molecule to split differentially across the biphase interface during lipid extraction.

To discover if the lipid fractions were capable of mediating responses of bovine innate immune cells, stimulation experiments were performed and the level of a range of cytokines was analysed. Significant increases in the production of various cytokines were measured only after cells were exposed to the polar lipid fraction. Perhaps most striking is the significant increase in the production of the Th-1 polarising IL-12 and the anti-inflammatory cytokine IL-10 (Figure 2a-b). While this may seem contradictory, it is important to note that the fractions used are complex mixtures of a variety of lipids some of which are known to induce potent immunostimulatory cytokine profiles, such as MMG [18] and others, such as glycerol monomycolate (GroMM) are known Th2 polarisers [31].

Little evidence exists on cytokine production by antigen presenting cells after treatment with lipids, however many lipids have been assessed in the context of both CD4^+^ and CD8^+^ T cells. For example, TDM, which is present in the polar and apolar fractions (Figure 1d-e), has been shown to induce both Th1 and Th2 cytokines. The induction of IFNγ and IL-12 and the depletion of IL-4 producing NK cells has been attributed to TDM [32,33] as well as a role, along with IL-6 and TNFα, in stable granuloma formation [34]. Yet TDM is also implicated in the production of IL-5 and IL-10 in a CD1 dependent manner [35]. Furthermore, GroMM has been implicated in the induction of Th2 polarising responses [31] where as the closely related GMM has been shown to induce Th1 cytokine responses in T cells [36]. Anti-inflammatory effects have also been attributed to PIM_2_ and PIM_6_ where, upon lipid treatment of LPS activated macrophages, Doz et al. measured downregulation of TLR4, TNFα, IL-12p40, IL-6, KC and IL-10 as well as MyD88 mediated NO release [37].

Given the significant increase in IL-10 production by all innate cell types assessed, and the important role these cells play in generating and directing the immune response, we analysed the expression of antigen presentation associated cell surface molecules after lipid exposure. Lipid treatment of APCs leads to a significant decrease in the levels of costimulatory molecules associated with antigen presentation including MHCII and CD86 on all cell types studied and CD1b on MDDC (Figure 3a-c). Negative regulation of these molecules by a variety of lipid components has been noted previously, especially MHCII in human and murine systems. Similar to the data presented here, the 19-kDa lipoprotein is capable of downregulating MHCII expression on human THP-1 macrophages by inhibiting activation of the IFNγ - induced CIITA [38,39]. Downregulation of MHCII, as well as TLR2 and TLR4, has also been reported on human MDDC after lipid exposure [40] and a further study also found impaired expression of CD1a, MHCII, CD80 and CD83 on human MDDC [41].

Downregulation of CD1 molecules has also been shown through the discovery that MDDC generated from BCG treated monocytes did not express CD1 and showed reduced MHCII, CD40 and CD80 [42] and this has since been shown to be due to cell wall associated carbohydrate α-glucan [43] and mediated through the p38 MAPK pathway [44]. However these experiments have all been performed in human or murine systems and with specific lipids, often from avirulent bacterial isolates.

Interestingly, treatment of fresh and cultured monocytes with the polar lipid fraction significantly increased the level of CD40 expression (Figure 3d) and this effect is not seen on MDDC. This finding seems contradictory to the published literature [42,45] however these studies used BCG or TDM alone, rather than the complex and more biologically representative lipid preparations derived from virulent mycobacteria used here, as well as being performed in human or murine macrophage models. Bovine MDDC expression of CD40 does not alter after stimulation with either polar or apolar lipids which may be due to its constitutively higher levels of expression than on fresh or cultured monocytes.

Finally, no difference was seen in the expression of CD80 after lipid treatment, although this has also been reported in other systems using virulent *M. tuberculosis* or avirulent BCG derived lipids [41,42,45].

The significant loss of MHCII, CD86 and CD1b is consistent with the phenotype of an impaired antigen presenting cell [46]. Given the effect of the polar lipids on the expression of these molecules and the concurrent increase in IL-10 production, we hypothesised that the polar lipid fraction, or one of its components, hampers the ability of the cells to successfully present antigen to T cells and may be able to suppress the induction of a Th1 response during infection. To assess any functional deficit in these cells, especially due to the loss in MHCII, lipid treated and untreated cells were used to drive allotypic proliferative responses.

Cultured monocytes drove proliferation of allogeneic PBMC (Figure 4) and treatment of cultured monocytes with the polar lipid fraction significantly abrogated these responses as suggested by the downregulation of MHCII and other costimulatory molecules. Proliferative responses were also seen when allogeneic PBMC were combined with untreated MDDC (Figure 4) however no difference in proliferation was seen using lipid treated MDDC despite flow cytometric analysis revealing characteristic reduction in the level of MHCII on the MDDC (data not shown). While these results seem at odds with each other, it is possible that the loss of MHCII may be overcome by the high level of CD40 expressed by MDDC (Figure 3d) or the constitutively higher levels of IL-12 produced by these cells which further increases significantly after lipid stimulation (Figure 2b). Also, some evidence exists that the presence of CD80 is enough to stimulate allogeneic T cells in the absence of CD86 signalling [47]. Given the significant reduction in CD86 expression on MDDC, the maintenance of CD80 may play a role. Finally, it is possible that, due to constitutively higher levels of MHCII and CD40 present on MDDC, as well as their expression of CD1b, the levels of MHCII and CD86 on these cells remains sufficient to drive an allotypic reaction.

These data demonstrate that *M. bovis* derived lipid fractions are capable of stimulating responses in bovine innate cells and that these different cell types respond in distinct ways.

Interestingly, the alteration in cell surface phenotype of both cultured monocytes and MDDC seen after polar lipid stimulation is also evident after exposure to the apolar lipid fraction, albeit to a lesser, not statistically significant, extent. This may be due to specific lipid components present in both the polar fraction and the apolar preparation, such as TDM. However, it may also be due to the insolubility of less polar lipids in the aqueous environment an in vitro culture system which may limit lipid bioavailability.

In conclusion, we present here the first data to demonstrate the regulatory effects of *M. bovis* - derived lipids on bovine innate cells. These lipids, especially those contained within the polar fraction are capable of interacting with the host’s innate immune cell’s such that the cells ability to initiate an adequate T cell response may be compromised, although this effect could only be demonstrated for cultured monocytes and not MDDC. The lipid fractions used in this study contain the total free extractable lipid from *M. bovis* AF 2122/97, hence we were not able to attribute these effects to any specific lipid entities. However work is currently being undertaken in our laboratory to further define these responses and identity the lipids which are responsible for mediating the effects we have shown. Nevertheless, the effects mediated by these lipids may play a pivotal role in the outcome of infection and aid further identification of individual lipid components responsible for the immunomodulatory effects as well as new targets for attenuation and novel vaccine candidates and adjuvant preparations.

## Competing interests

The authors declare that they have no competing interests.

## Authors’ contributions

CP - Carried out the studies and prepared the manuscript. SS - Assisted with dendritic cell culture methods. GJ - Participated in the study design and proofing of the manuscript. GB - conceived of the study and participated in its design and coordination. MV - conceived of the study and participated in its design and coordination and helped to draft the manuscript. All authors read and approved the final manuscript.
